# A designathon to co-create HPV screening and vaccination approaches for mothers and daughters in Nigeria: findings from a community-led participatory event

**DOI:** 10.1186/s12879-024-09479-7

**Published:** 2024-06-20

**Authors:** Eneyi E Kpokiri, Agatha E Wapmuk, Chisom Obiezu-Umeh, Ucheoma Nwaozuru, Titilola Gbaja-Biamila, Ifeoma Obionu, Ekenechukwu Kokelu, Jennifer Smith, Benedict N Azuogu, Kayode Ajenifuja, Abdulhammed O Babatunde, Oliver Ezechi, Joseph D Tucker, Juliet Iwelunmor

**Affiliations:** 1 Clinical Research Department, Faculty of Infectious and Tropical Diseases, London School of Hygiene and Tropical Medicine, London, UK; 2 Nigerian Institute of Medical Research, Lagos, Nigeria; 3 Department of Behavioral Science and Health Education, Saint Louis University, Saint Louis, MO USA; 4 Department of Implementation Science, Wake Forest School of Medicine, Winston-Salem, NC USA; 5 Division of Infectious Diseases, School of Medicine, Washington State University, St Louis, MO USA; 6 Institute of Global Health and Infectious Diseases, University of North Carolina at Chapel Hill, Chapel Hill, NC USA; 7 Department of Anaesthesia, Ebonyi State University, Abakaliki, Ebonyi State Nigeria; 8 Department of Obstetrics and Gynaecology, Obafemi Awolowo University, Ile-Ife, Osun State Nigeria; 9 Departmrnt of Medicine and Surgery, Faculty of Clinical Sciences, College of Medicine, University of Ibadan, Oyo, Nigeria

**Keywords:** HPV, Cervical cancer, Designathon, Community-led, Nigeria

## Abstract

**Background:**

Oncogenic types of human Papillomavirus (HPV) infection cause substantial morbidity and mortality in Nigeria. Nigeria has low cervical cancer screening and vaccination rates, suggesting the need for community engagement to enhance reach and uptake. We organised a designathon to identify community-led, innovative approaches to promote HPV screening and vaccination for women and girls, respectively, in Nigeria. A designathon is a three-phase participatory process informed by design thinking that includes the preparation phase that includes soliciting innovative ideas from end-users, an intensive collaborative event to co-create intervention components, and follow-up activities.

**Methods:**

We organised a three-phase designathon for women (30-65yrs) and girls (11-26yrs) in Nigeria. First, we launched a national crowdsourcing open call for ideas on community-driven strategies to support HPV screening among women and vaccination among girls. The open call was promoted widely on social media and at in-person gatherings. All eligible entries were graded by judges and 16 exceptional teams (with 4-6members each). All six geo-political zones of Nigeria were invited to join an in-person event held over three days in Lagos to refine their ideas and present them to a panel of expert judges. The ideas from teams were reviewed and scored based on relevance, feasibility, innovation, potential impact, and mother-daughter team dynamics. We present quantitative data on people who submitted and themes from the textual submissions.

**Results:**

We received a total of 612 submissions to the open call from mother-daughter dyads. Participants submitted ideas via a website designated for the contest (*n* = 392), in-person (*n* = 99), email (*n* = 31), or via an instant messaging application (*n* = 92). Overall, 470 were eligible for judging after initial screening. The average age of participants for daughters was 19 years and 39 years for mothers. Themes from the top 16 proposals included leveraging local leaders (5/16), faith-based networks (4/16), educational systems (4/16), and other community networks (7/16) to promote awareness of cervical cancer prevention services. After an in-person collaborative event, eight teams were selected to join an innovation training boot camp, for capacity building to implement ideas.

**Conclusions:**

Innovative strategies are needed to promote HPV screening for mothers and vaccination for girls in Nigeria. Our designathon was able to facilitate Nigerian mother-daughter teams to develop cervical cancer prevention strategies. Implementation research is needed to assess the effectiveness of these strategies.

**Supplementary Information:**

The online version contains supplementary material available at 10.1186/s12879-024-09479-7.

## Introduction

Cervical cancer causes substantial mortality and morbidity in Nigeria. Cervical cancer is the second most common cancer among women in Nigeria. Each year in Nigeria, 12,000 women are diagnosed with, and 7,800 women die from this highly preventable cancer [1, 2]. However, Human Papillomavirus (HPV) screening with the treatment of precancerous lesions and vaccination are effective in preventing cervical cancer. Challenges exist in ensuring all women who screen HPV-positive are retained in care and treated. In Nigeria, HPV vaccination among girls and cervical cancer screening in women remains low [3–5]. A review showed an overall pooled estimated HPV vaccine uptake of 28.53% among adolescent schoolgirls in sub-Saharan Africa, while in Nigeria, HPV vaccination rates for adolescent girls stood at 1.4% [6]. This is much higher in the United States where HPV vaccination rates for schoolgirls aged 13-17yrs is 65.1% [7]. At the time of conducting this study, HPV vaccination access in Nigeria is available via to out-of-pocket purchase in private health-care facilities. Many women and girls in Nigeria have limited awareness of cervical cancer and methods for prevention [8, 9]. This contributes to the low uptake rates in addition to the high price of obtaining the vaccine and low health insurance coverage in Nigeria [10]. These are similar factors noted for low uptake in Africa and other LMICs [11–13]. 

Engaging the local community to identify and co-create strategies to increase HPV vaccination and cervical cancer screening can be an effective strategy to improve awareness. Participatory approaches such as crowdsourcing open calls and designathons have been used to increase community engagement to co-create interventions [14, 15]. Crowdsourcing has a group contribute ideas to solve all or part of a problem and then share selected solutions widely with the public [16, 17]. Crowdsourcing also referred to as co-production or co-creation has been used to generate innovative ideas, logos, images, videos, design clinical prevention services, or new technologies to improve health outcomes [18]. A designathon is a three-stage participatory activity informed by design thinking that includes preparation with end-users, an intensive period of collaborative team work, and follow-up activities for implementation and research [19, 20]. Designathons have been used to refine and finalize interventions in diverse settings [21, 22]. In Nigeria, designathons have been used to co-create community-driven HIV self-testing services for Nigerian youth [22, 23]. 

Following the need to increase awareness and engagement, we organised a designathon to engage mother/daughter teams to develop community-led strategies to increase HPV vaccination among girls and HPV screening among women. The purpose of this paper is to describe the designathon process, summarize the community-led strategies proposed and discuss public health implications.

## Methods

**Fig. 1:**
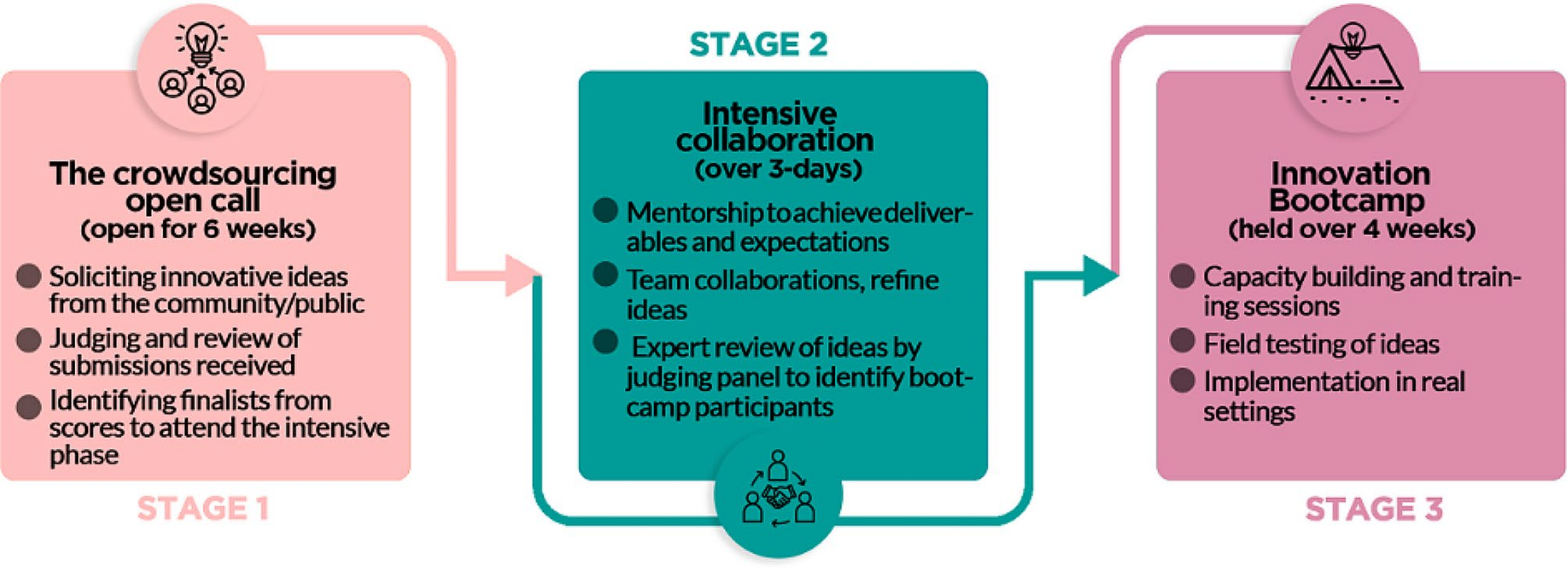
Overview of the key stages employed for the Designathon process

These participatory activities were organized as part of a larger study called the Actions for Collaborative Community-Engaged Strategies for HPV (ACCESS-HPV) (R01CA271033). We decided to focus on mother/daughter dyads given that the engagement of those directly receiving cervical cancer prevention interventions may increase the likelihood of the future uptake of participatory strategies. It capitalizes on reciprocal learning between mothers and daughters and reinforces prevention attitudes and behaviours. Dyadic strategies align with cultural values in Nigeria where family involvement in health decisions is a common expectation. The process started with a national crowdsourcing open call for ideas from teams comprising mother and daughter dyad teams, then followed an intensive period of collaboration to allow the teams to refine and pitch ideas and finally, an innovation training boot camp for capacity building to support implementation of the ideas (Fig. 1). For the crowdsourcing open call, we followed the steps outlined in the WHO/TDR/SESH/SIHI practical guide on crowdsourcing in health and health research [16]. The designathon followed steps outlined in the WHO/TDR/SESH/SIHI practical guide on designathons [20]. The overall purpose of the designathon was to finalize a set of community-engaged HPV screening and vaccination interventions, identify local female leadership for implementation, and to galvanize momentum for policy action.

### The crowdsourcing open call

The crowdsourcing open call was launched on 1st February 2023 and remained open until 12th March 2023. A website was created and hosted on the for the girls and women (4GW) online page and contained detailed information about the call for ideas including guidelines for participation and an online submission platform. The link to the website was shared widely as through social media channels, women’s networks, youth groups, and some in-person meetings. The members of the steering committee also disseminated the call for ideas through their networks. Dyadic teams of mothers and daughters could submit online (website, email or WhatsApp) or offline (paper copies, in-person). At the end of the promotion, all entries received were screened for eligibility by the core team. Eligible entries were then put in sets and sent to pre-identified volunteer judges for reviews. To ensure fairness in reviewing submissions, the judges were provided with a judging rubric to guide allocation of scores. (Appendix I). Scores received from the judges were analysed and top-ranking finalist teams were invited to join the intensive collaborative phase.

### The intensive collaborative phase

The in-person intensive collaboration part of the designathon was organized between 31st March and April 2nd, 2023, in Lagos Nigeria, where 16 teams selected from the national crowdsourcing open call with exceptional ideas to increase HPV screening and vaccination attended. All attendees were assigned key roles as participants, mentors/facilitators, judges or organizers. A handbook was developed outlining the purpose of the event and expectations across the different roles. (Appendix II) The participants were asked to bring their creative ideas to the event for further development and refining, while the mentors/facilitators were to provide guidance and feedback based on their expertise. The judges were sector leaders and experts from across Nigeria to determine feasibility and potential of the ideas to generate impact. The organisers were a group of dynamic global health experts overseeing the process and providing support to the teams to ensure that they understand the goals, structures and stay on task at all stages. During the intensive collaboration phase, the teams received guidance on the expectations and mentorship to achieve the deliverables. They refined their ideas and made an oral pitch presentation of their refined ideas to an expert judge panel.

On Day 1 of the intense collaborative phase, the teams received further details on the purpose of the designathon, the rules and other expected deliverables over the three-day period of collaborations. The designathon deliverables included a working prototype/user journey and an executive summary of the ideas, a five-minute pitch and team photos. Participants also received short talks on cervical cancer and HPV.

Day 2 was marked for rapid prototyping, expert feedback and iterations. Here the teams built on insights and inspirations from the sessions on Day 1, refined and strengthened their ideas through discussions and feedback from experts and advisory mentors. At the end of day 2, participants were given the option to do a practice run of their pitches with mentors available to provide feedback.

Day 3 saw the final presentations of pitches put together by the teams, explanations of the ideas and user journey to a panel of judges. Judges included experts in HPV and cervical cancer services, advocates, community leaders, religious group representatives and adolescent service providers. After judging, the top three teams were announced and cash prizes were provided with first place receiving N500,000 (USD 634), second place receiving N350,000 (USD 443) and N250,000 (USD 317) for third place. The pitches were judged by a pre-specified criteria which including innovation, relevance, feasibility, teamwork and promotion of equity and fairness. Innovation referred to how novel and unique is the proposed solution, relevance explored how relevant the idea will be for a global majority in the target age group of a given intervention (female adolescents for HPV vaccination; women aged 25 and older for screening. Feasibility referred to how easily the ideas can be implemented in diverse Nigerian settings. The teamwork criteria assessed ability of participants working in teams, delegating tasks, utilizing strengths and working together. Finally, the ideas were scored on potential to address issues of equity and fairness.

### The follow-up activities

The follow-up activities included an innovation bootcamp lasting 4 weeks (9th June to 11th July 2023). The participants had intensive training sessions from experts around project logistics and implementation science. Specific topics include budgeting and logistics management, storytelling, human centred design, research skills (qualitative and quantitative methods), and strength, weakness, opportunities and threats (SWOT) analysis reviews. While week one focused on the lectures, week two was dedicated to skills building and practical application of the lessons received in week one. The third week saw participants going out into the communities for field testing, meeting with and community partners; in-depth field observations of potential sites for pilot studies. The fourth and final week was set aside for group work, putting it all together and preparations of pitch and final presentations.

All participants were provided with accommodation, food and local transportation during their stay for the intense collaboration and bootcamp stages of the designathon. The different teams were provided with colour coded T-shirts and a folder containing the Designathon handbook, writing materials and other items needed. General rules for the participants include ideas presented must be original works, no plagiarism, all participants should be present and actively participating during the key stages of the Designathon and respect the instructions set by the organisers and programme coordinators.

### Data analysis

**Fig. 2:**
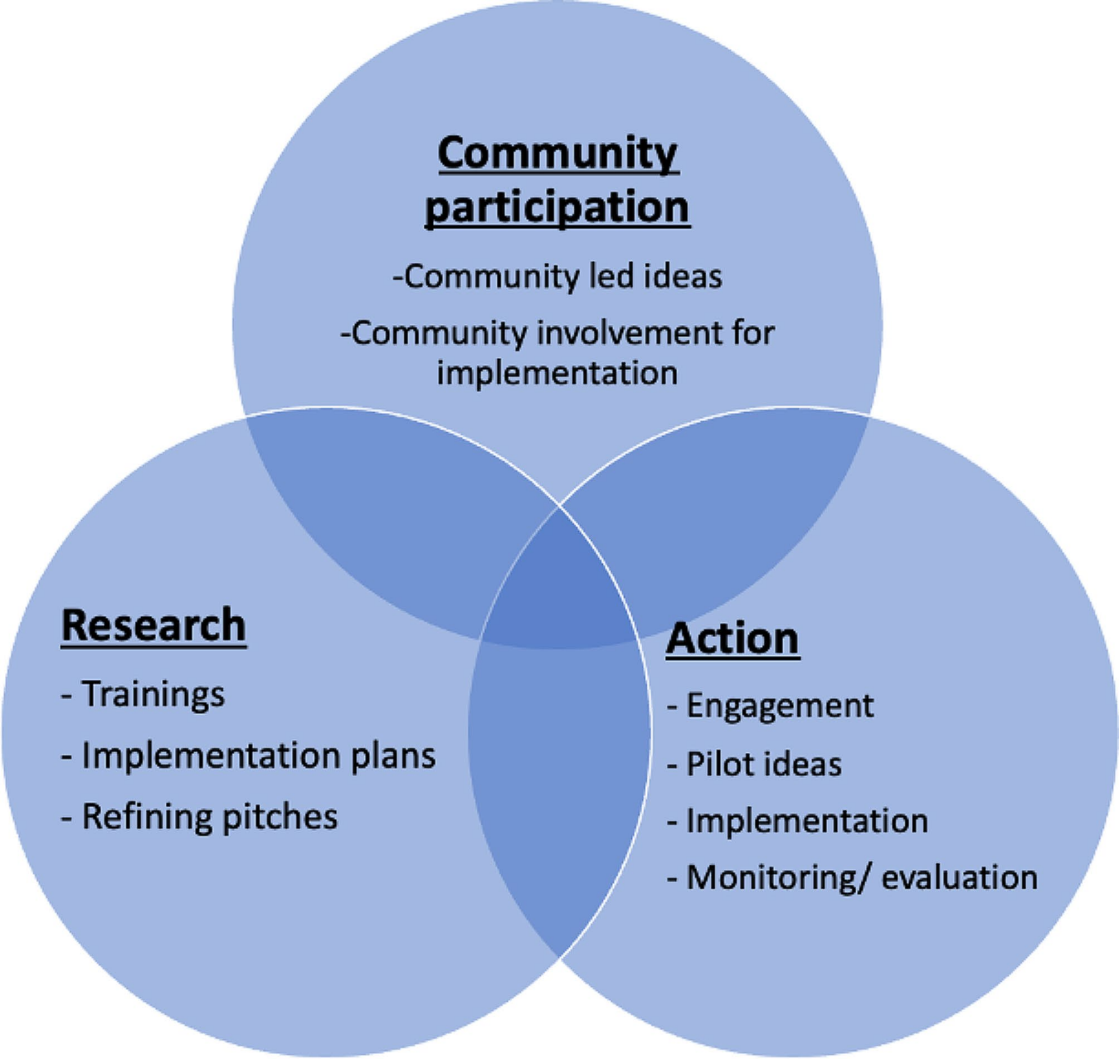
Framework for participatory action research (PAR)

We organised and presented the participant characteristics and demographic data using descriptive statistics. The ideas of the exceptional teams were presented in the terms of location, team dynamics, focus of the approach and intervention components. We organised the results of the Designathon following broad domains included in the framework of participatory action research (PAR). This includes community participation, research, and action (Fig. 2).

## Results

**Fig. 3:**
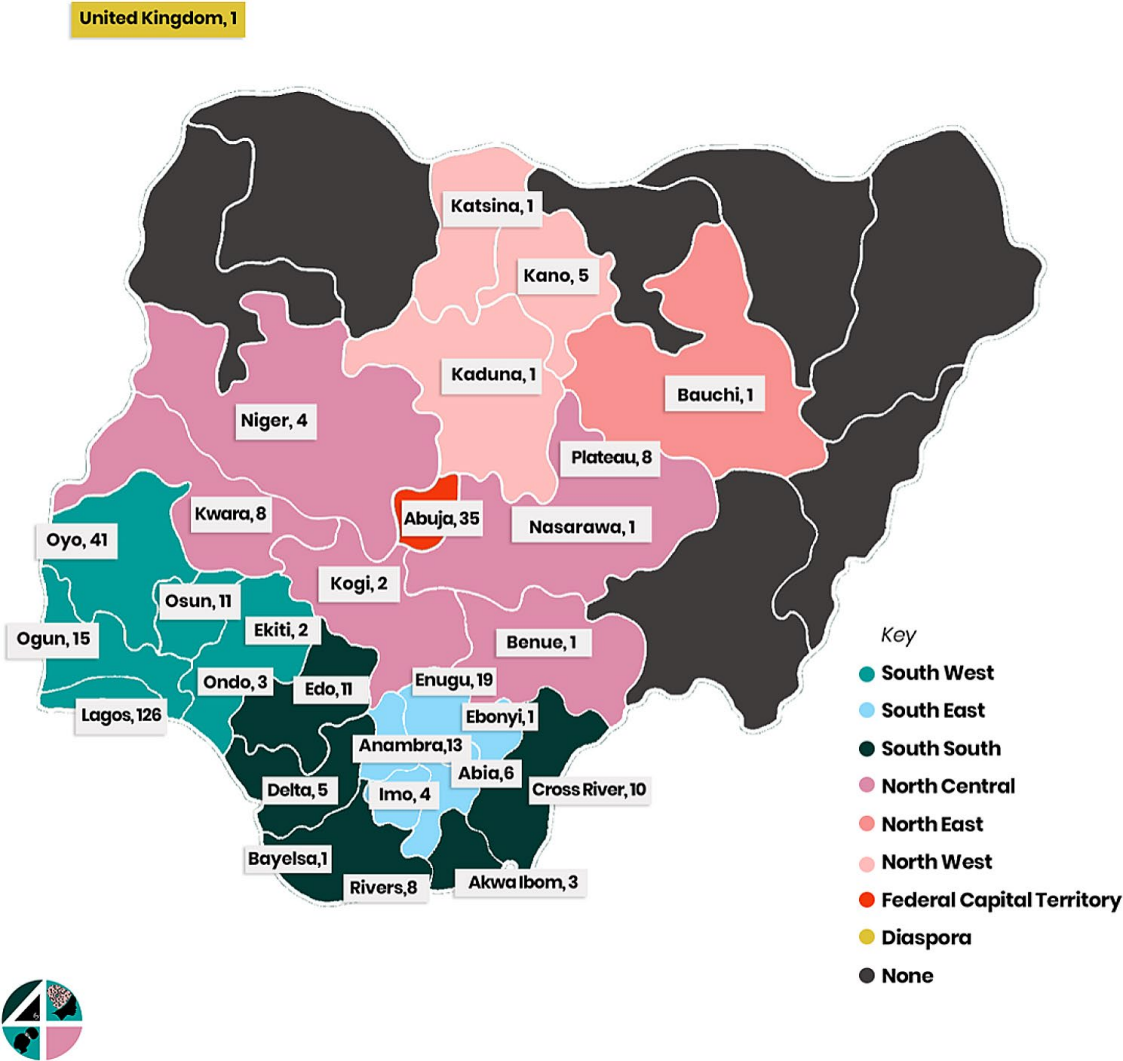
Distribution of submissions received by state in Nigeria

The crowdsourcing open call for ideas received a total of 612 submissions to the open call, entries were received by online submissions (*n* = 392), in-person submissions (*n* = 99) and via the WhatsApp instant messaging application (*n* = 92) and from emails (*n* = 31). From this total, 470 were eligible for judging. Submissions received to the open call was widespread across Nigeria (27/36 states) (Fig. 3). From the six geo-political zones in Nigeria, most submissions came in from the South-West regions, six states with Lagos and Oyo states having the highest numbers (*n* = 126 and 41 entries respectively). Abuja being the Federal Capital Territory (FCT) had the third highest number of entries (= 35) and we received one submission from a Nigerian based in the United Kingdom.

A total of 73 participants across 16 teams were selected from participants of the crowdsourcing open call to join in the second phase of the Designathon (Table 1). The average age of participants for daughters was 19 years and 39 years for mothers. All eligible entries were graded by judges and 16 exceptional teams (with 4-6members each) from all six geo-political zones of Nigeria were invited to join the Designathon event. Themes from proposals included leveraging local leaders (five strategies), faith-based networks (four strategies), community networks (seven strategies) educational systems (four strategies) and annual female-related celebrations (two strategies). After three days of developing their proposals and a pitch presentation to the judging panel, eight teams, including three finalists were selected to join the proposed innovation boot camp.

**Table 1:** Overview of top-ranked teams from the 2023 “4 Girls and Women” HPV vaccination and HPV screening designathon

S/*N*	Team Name (Location)	Composition Number (ages in yrs)	Focus of Approach	Intervention components
1.	*Camgo (Oyo State)	4 (55, 28, 25, 11)	Individual, social, community	Faith-based approach via Redeem church network, Coalition with cervical cancer survivors, will be leveraged as locations for Mother and child to pick up CAMGO Bags which has the self-test kit to screen and vaccination for young girls. And as a Location to drop self-test kits for dispatch to assessment and result disclosure to women
2.	*Branden (Ogun state)	4 (37, 23, 23, 16)	Individual, social, community	A community-based approach to promote awareness about cervical cancer and its preventive measures, and improve accessibility, availability, and acceptability.
3.	*Shade (Akwa Ibom)	4 (41, 25, 24, 23)	Individual, social, community	Community-based sensitization via radio, market campaigns and social media
4.	*White (Lagos)	5 (57,50,49,21,21)	Social, community	Demand creation with the National Youth Service Corporation, advocacy with relevant stakeholders
5.	*Conquerors (Niger)	4 (37, 32, 11, 26)	Individual, social, and community	Schools and Faith-based organizations to disseminate screening kits and vaccination schedules/information
6.	*ReachHer (Bayelsa)	4 (31, 31, 26, 23)	Individual, social, community	On-site vaccination of girls, and distribution of self-sampling kits, use of mobile screening booths
7.	*Girlcare (Calabar)	6 (30,30,24,23,23,20)	Individual, community	Pharmacy-based services, digital tools to enable internet users to access locations for vaccination and screening.
8.	*EmoOghene (Yola)	4 (50, 48, 21, 16)	Individual, social, community	Faith-based approach, partnerships with men, mini-diary campaign on HPV services
9.	Charis (Bauchi & Abuja)	4 (33, 26, 25, 24)	Individual, social, community	Community based sensitization, provision of direct help lines, provision of a safe space for HPV screening and vaccination
10.	Soar (Jos)	4 (34, 26, 26, 25)	Individual, social, community	Community based approach, use women organizations, involve local influencers to promote HPV vaccination and HPV screening. Use popular comedians to promote HPV vaccination and screening in women
11.	Agojie (Lagos)	5 (52,24,24,22,22)	Individual, social, community and school	Community based approach, school-based approach, use of market leaders for market campaigns and M- health
12.	Witty (Enugu)	4 (32, 30 28, 24)	Individual social community	Community based approach involving sensitization and community mobilization using community gate keeper
13.	Salu-Lawa (Katsina)	6 (60,30,29,28,18,11)	Individual, community	Faith based leveraging on the Islamiyah school to disseminate information and increase knowledge and awareness about HPV and distribute test kits.
14.	Female Health matters (Enugu)	4 (32, 28, 25, 24)	Community, social	Leveraging on existing NGOs working on cervical cancer prevention and prevention of other diseases like HIV. They will collaborate to integrate HPV vaccination for girls and screening for women into the NGOs services
15.	Heroes for her (Ibadan, Abuja, Anambra)	4 (30, 26, 25, 23)	Individual, social, community	Community based sensitization using radio, road walks, radio, market campaigns with market leaders and social media, all in partnership with the local government operated health centres
16.	HPV Crusaders (Abia)	4 (34, 32, 28, 11)	Individual, community	Community mobilization using community gate keepers, women organization leaders, faith-based approach

*Top 8 teams from the Designathon, selected to attend the Innovation Boot Camp

### Team compositions and HPV services

All teams had representatives of mothers and daughters and had total team numbers ranging from four (lowest) to six (highest). All the proposals from the 16 teams selected to attend the intensive collaboration phase included strategies to spur uptake of HPV vaccination for girls and screening for women. This was a requirement for submissions sent in through the crowdsourcing open call.

### Engagement

The focus of the approach presented within the proposals from the 16 finalist teams were broadly classified into individual, social and community focus. Some had a combination of all three (8/16 strategies), some had a combination of social and community focus (2/16 strategies) and individual and community (6/16 strategies).

### Implementation strategies

Majority of the implementation strategies relied heavily on community networks. This includes faith-based approaches (5 strategies), local leaders and notable people (7 strategies), markets (3), and mobile, media and tele-health approaches (7 strategies). These networks were largely leveraged to generally improve knowledge and awareness about HPV and cervical cancer within the communities and to increase access to tests kits for screening and vaccination. In addition, the local media such radio and television stations, market campaigns and social media jingles were proposed as potential channels to disseminate the HPV services (6 strategies). While faith-based institutions, market stalls, community pharmacies and schools will be used to distribute test kits for screening and vaccination programmes within the communities (8 strategies).

## Discussion

We organised and implemented a designathon in which we crowdsourced ideas on strategies to support HPV vaccination for girls and cervical cancer screening for girls and women in Nigeria. This provided a unique opportunity for girls and women to collaborate and work together in daughter- mother teams and develop these ideas. We extend the literature by using a crowdsourcing and designathon methodology to build capacity and identify HPV screening/vaccination implementation strategies.

Our study findings suggest mother-daughter dyadic collaborations was effective to co-create and disseminate strategies for the uptake of HPV vaccination and cervical cancer screening services. This approach leverages reciprocal learnings between the mothers and daughters, which is more likely to reinforce behaviour changes associated with health prevention approaches. Community-based research studies have also demonstrated that mother/daughter interventions that provide vaccination to young girls and self-collection to their mothers are feasible and effective [24]. Working in mother-daughter dyad collaborations for HPV and cervical cancer also aligns with the cultural norms in low income settings where girls are seen to largely defer to their mothers/mother figures in issues regarding their sexual and reproductive health [25]. 

This series of participatory activities resulted in high-quality strategies as determined by independent volunteer judges. This is consistent with earlier studies that reported crowdsourcing and designathons leveraging strengths of diverse groups in the community to co-create health interventions [21, 26, 27]. Crowdsourcing is an innovative bottom-to-top problem-solving approach that harnesses the collective intelligence and diverse skills of a large community, known as the ‘crowd’. Bottom-up approaches are more likely to generate innovative and impactful solutions with greater uptake with greater potential for sustainability in the long term. There have been several randomized control trials conducted to evaluate the impact and effect of crowdsourced interventions that found them to be more effective than conventional approaches or standard of care [28, 29]. The local resources and local networks are often being tapped into for actualizing the ideas, and thereby increasing the acceptability and relevance within the local settings. Given the higher acceptability and sustainability of crowdsourced interventions, these strategies have been employed for other complex health problems such as HIV testing and diagnosis [14], alcohol reduction [30] and obesity prevention [31]. 

Several of the innovative community-led ideas proposed by the teams focused on the individual and communities, while some had a social focus. Most ideas leveraged community networks including faith-based organisations, local community leaders, schools and related national celebrations. Nigeria as a country embeds one of its core values in religious worship and can be leveraged for dissemination of services across other sectors including health. This can inform future work to engage communities of interest such as faith-based organizations for health programs. Given the knowledge base in Nigeria for HPV and cervical cancer has been so low, integrated services with existing structures will potentially increase acceptability and uptake and may prove to be an effective approach to reach women and girls [15, 22, 23]. 

Our study had some limitations as the ideas selected for implementation were solely judged as feasible by the expert panel, based on the team pitches, there were no outcome or pilot data. The series of participatory events were held at different time points over varying durations. The intensive collaboration and bootcamp sessions were in-person and affected team dynamics as not all team members were readily available to attend continuously and consistently. The phase 2 with intensive collaboration had teams working rapidly to prototype their ideas, refine and pitch proposals over a tight timeline of 72 h. However, following selection, the exceptional ideas and teams were invited to an intensive 4-week innovation bootcamp to build capacity and enhance skill of the teams for implementing their ideas in real settings, providing mentorship in implementation science as well to the teams. Until now, low levels of cervical cancer knowledge and awareness have been reported across Nigeria [32–34]. Our study with a series of participatory activities for HPV and cervical cancer with intense publicity and dissemination across multiple social media platforms, this work ultimately increased the knowledge and awareness base about cervical cancer within local communities in Nigeria (Appendix II). To prevent loss to follow up for women who screen positive, all sites have been linked with teaching hospitals and gynaecologists who will serve as site supervisors. All institutions will follow the Society of obstetrics and gynaecology of Nigeria–Clinical practice guidelines (SOGON) guidelines for follow up care [35]. 

While we have been able to engage females in Nigeria to locally co-created HPV and cervical cancer screening services for implementation in their local communities, there is need for further research to further explore the impact of these strategies and their sustainability in the longer term.

### Electronic supplementary material

Below is the link to the electronic supplementary material.

Supplementary Material 1

## Data Availability

The datasets used and/or analysed during the current study available from the corresponding author on reasonable request.
