# Supplementary material for: A designathon to co-create HPV screening and vaccination approaches for mothers and daughters in Nigeria: findings from a community-led participatory event
Source: BMC Infect Dis. 2024 Jun 20;24:606. doi: 10.1186/s12879-024-09479-7 (PMC11188243; doi:10.1186/s12879-024-09479-7)
Supplement: Supplementary file 1 — Supplementary Material 1 [file 12879_2024_9479_MOESM1_ESM.docx]

**Supplemental files**

**Appendix 1: Open Call Judging Rubric**

**Appendix II: Engagement Data and SOCIAL MEDIA REPORT**

**Appendix III: The designathon Handbook**

**Appendix IV: Sample of the open call promotion banner**

**Appendix 1: Open Call Judging Rubric**

**4 Girls & Women (4GW) Idea Contest 2023**

Judging Criteria and Instructions

Thank you for serving as a judge for the 4GW Open Contest. We greatly appreciate your time and effort in supporting this initiative. For more information on the open call, please visit 4GW.org. Please complete your evaluations and send your scores by **11:59 pm WAT (5:00 pm CST) on Thursday, March 16, 2023**. We anticipate that scoring submissions will take 2-3 hours to complete. Please do let us know if you will be needing more time. There are three phases to the judging process:

- Phase I: Pre-screening of submissions for eligibility by the 4GW study team.
- Phase II: Evaluation of eligible submissions by the 4GW judging team, based on a scale of 1-3 per criteria.
- Phase III: External panel of Judges will determine finalists at the Designathon.

**Instructions:** This is phase II of the judging process, to be completed by members of the judging team. You will be evaluating each submission based on the judging criteria below. Please provide a score on a scale of 1 to 3 (with 1 being the poorest quality and 3 being the highest quality) for each criterion. During your evaluation, please focus on the content of the submissions. The total of each individual criterion will be computed. Scores for each entry will be averaged and entries will be ranked Each individual submission will be scored by 3 judges. The scores for each submission will be tallied, averaged, and then ranked. We will provide a comments column for positive and negative comments if you wish to add any additional details to your score. We have de-identified the submissions for the evaluation process.

**Judging Criteria:**

1. **Relevance**

- This refers to whether the idea is relevant to mothers/daughters in the Nigerian context.

1. **Innovation**

- To what extent is the submission unique, innovative, and novel?
- Does the submission describe new opportunities to promote HPV vaccination and screening in Nigeria?

1. **Feasibility of implementing the solution**

- Feasibility refers to how easily and practically implementable the innovation would be in diverse Nigerian settings.
- Would it work in rural and/or urban settings in Nigeria?

1. **Equity**

- Does the idea empower girls and women to vaccinate or screen for HPV?
- How can we inspire men to be allies?

**Scoring Guide:**

| Score | Description: |
| --- | --- |
| 4 | Submission meets none of the criteria (it is not clearly presented, not relevant, not innovative, not feasible, and has no potential for a positive impact on HPV vaccinations for girls and HPV screening for women in Nigeria. |
| 5 – 6 | Submission meets some of the criteria, but weakly. |
| 7 – 8 | Submission meets some of the criteria, with moderate strength. |
| 9 – 10 | Submission meets all of the criteria, with moderate strength. |
| 11 - 12 | Submission meets all of the criteria, with exceptional strength (very clearly presented, highly relevant, extremely innovative, highly feasible, and has exceptional potential for positive impact on HPV vaccinations for girls and HPV screening for women in Nigeria). |

**Appendix II: Engagement Data and SOCIAL MEDIA REPORT (21ST Feb. TO 22ND August 2023)**

| Social Media Platform | Instagram | Twitter (X) | LinkedIn | Facebook |
| --- | --- | --- | --- | --- |
| Data received | Followers:178 - 1,085 (6x)  New followers: 907  72 feed Posts (15 Reels)  Reach: 53K | Tweets: 132  Impressions: 18.5k  Followers: 17 to 80 (63 new followers)  Highest: 358 (who is eligible to participate, 27 Feb) | Followers: 65 to 249 (3.8x)  New followers: 184  Page visitors: 165 (371.4% increase)  Contents reactions: 383  Impressions: 5.3k | Post reach: 357  Post engagement: 700  Likes: 11  New followers: 25 |

**Remark**

The Instagram account has achieved significant growth over the last 6 months while the LinkedIn and Twitter fairly grew and Facebook has the least growth. The social media need to sustain the growth on Instagram and focus on also increasing engagement on the platform. New strategies will be implemented to increase growth of other social media pages.

Some notable challenges include

- Twitter: Change in Twitter algorithm to limit the reach of unverified accounts and users love to engage controversial posts the most
- Instagram: Inappropriate post time
- Facebook is mostly used for sponsored ads now and most young Nigerians have abandoned the platform
- LinkedIn: Users engage professional opportunities and career development

**Plan**

- Twitter: engage with influencer posts and use of trending hashtags. Organise monthly twitter space webinar on relevant topics and invite influencers
- Instagram: create contents early for early approval and appropriate post time. Engage more with Nigerian contents and share funny memes and life hacks on stories. Celebrate influencers through personality of the month to get reposted. Organize competitions and quiz.
- LinkedIn: Invite more followers. Create posts targeting career development and opportunities in health research and innovation.
- Facebook: Ensure to always posts contents on Linkedin also on facebook.

**Appendix III: The designathon Handbook available here:**

[***https://drive.google.com/drive/search?q=Designathon%20Handbook***](https://drive.google.com/drive/search?q=Designathon%20Handbook)

**2**

**Appendix IV: Sample of the open call promotion banner**

**
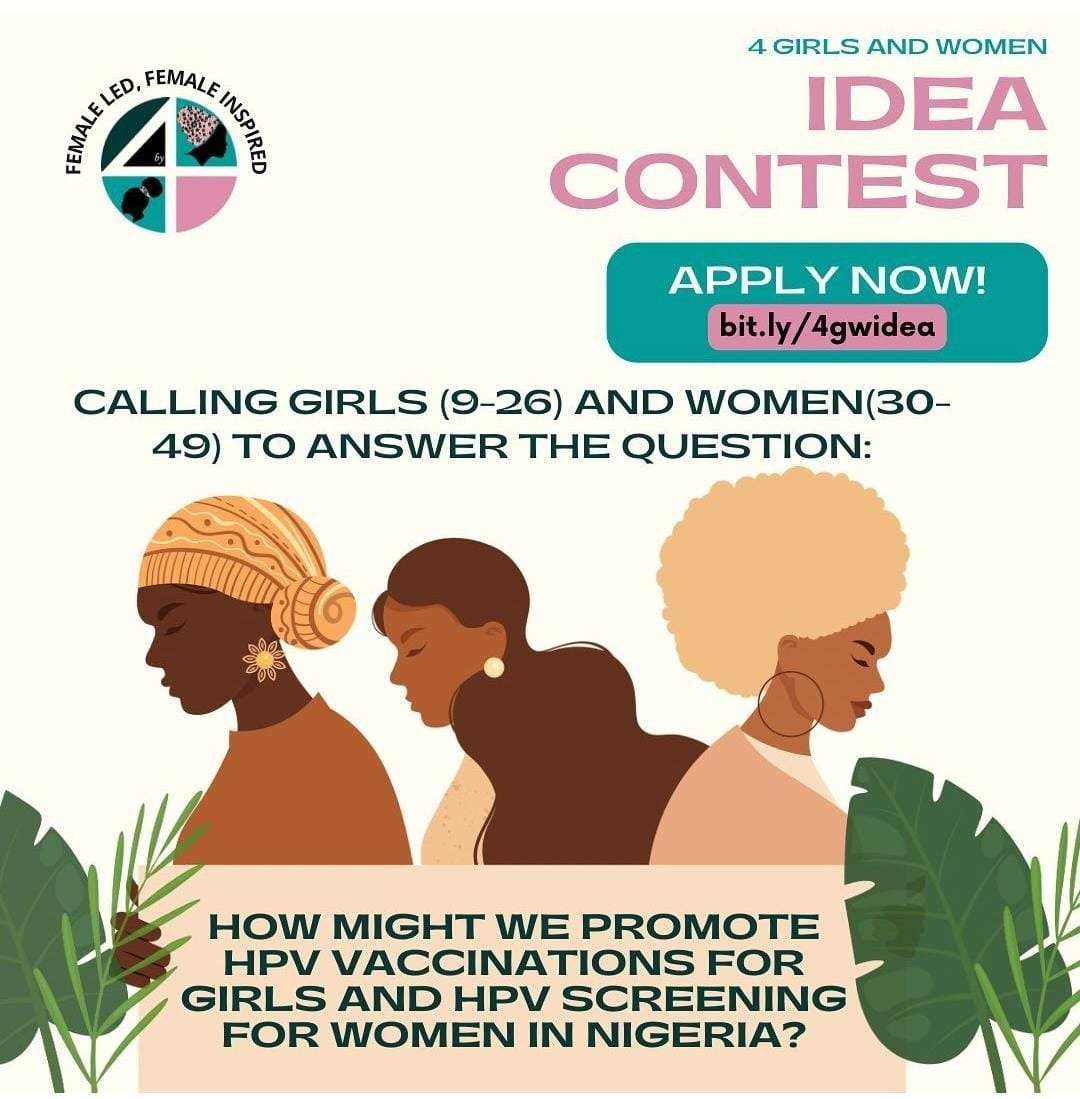
**
